# Ventilatory and Autonomic Regulation in Sleep Apnea Syndrome: A Potential Protective Role for Erythropoietin?

**DOI:** 10.3389/fphys.2018.01440

**Published:** 2018-10-16

**Authors:** David C. Andrade, Liasmine Haine, Camilo Toledo, Hugo S. Diaz, Rodrigo A. Quintanilla, Noah J. Marcus, Rodrigo Iturriaga, Jean-Paul Richalet, Nicolas Voituron, Rodrigo Del Rio

**Affiliations:** ^1^Laboratory of Cardiorespiratory Control, Department of Physiology, Pontificia Universidad Católica de Chile, Santiago, Chile; ^2^Centro de Investigación en Fisiología del Ejercicio, Facultad de Ciencias, Universidad Mayor, Santiago, Chile; ^3^Laboratoire Hypoxie and Poumon – EA2363, Université Paris 13, Paris, France; ^4^Centro de Envejecimiento y Regeneración (CARE), Pontificia Universidad Católica de Chile, Santiago, Chile; ^5^Centro de Investigación Biomédica, Universidad Autónoma de Chile, Santiago, Chile; ^6^Department of Physiology and Pharmacology, Des Moines University, Des Moines, IA, United States; ^7^Laboratorio de Neurobiología, Department of Physiology, Pontificia Universidad Católica de Chile, Santiago, Chile; ^8^Centro de Excelencia en Biomedicina de Magallanes (CEBIMA), Universidad de Magallanes, Punta Arenas, Chile

**Keywords:** erythropoietin, peripheral chemoreflex, central chemoreflex, chronic intermittent hypoxia, sleep apnea

## Abstract

Obstructive sleep apnea (OSA) is the most common form of sleep disordered breathing and is associated with wide array of cardiovascular morbidities. It has been proposed that during OSA, the respiratory control center (RCC) is affected by exaggerated afferent signals coming from peripheral/central chemoreceptors which leads to ventilatory instability and may perpetuate apnea generation. Treatments focused on decreasing hyperactivity of peripheral/central chemoreceptors may be useful to improving ventilatory instability in OSA patients. Previous studies indicate that oxidative stress and inflammation are key players in the increased peripheral/central chemoreflex drive associated with OSA. Recent data suggest that erythropoietin (Epo) could also be involved in modulating chemoreflex activity as functional Epo receptors are constitutively expressed in peripheral and central chemoreceptors cells. Additionally, there is some evidence that Epo has anti-oxidant/anti-inflammatory effects. Accordingly, we propose that Epo treatment during OSA may reduce enhanced peripheral/central chemoreflex drive and normalize the activity of the RCC which in turn may help to abrogate ventilatory instability. In this perspective article we discuss the potential beneficial effects of Epo administration on ventilatory regulation in the setting of OSA.

## Introduction

Sleep apnea (SA) syndrome is a pathological condition characterized by recurrent airway obstruction or cessation of breathing during sleep (Gislason et al., 1988; Epstein et al., 2009; Dempsey et al., 2010) resulting in hypercapnia and hypoxemia/oxyhemoglobin desaturation (Badran et al., 2014a). The collapse of the upper airway during obstructive events is likely attributable to both anatomical and non-anatomical determinants (Dempsey et al., 2010) including changes in central and peripheral respiratory drive (Solin et al., 2000; Kara et al., 2003; Dempsey et al., 2012).

Acutely, activation of peripheral and/or central chemoreflexes during apneic episodes is associated with micro-arousals and sleep fragmentation, as well as increases in ventilation, sympathetic nerve activity, blood pressure, and heart rate. Repetitive apneas as observed in SA syndrome are associated with increased diurnal drowsiness, neurocognitive dysfunction, and cardiovascular morbidity (Young et al., 2002; Gozal, 2013; Konecny et al., 2014). In clinical populations, cardiovascular morbidity in SA patients is often associated with enhanced activity of the sympathetic nervous system, and numerous studies indicate that this increased sympathetic activity stems from a heightened carotid body chemoreflex (CBC) (Narkiewicz et al., 1998; Mansukhani et al., 2015; Iturriaga, 2017). In addition to affecting autonomic outflow, aberrant CBC activity may also have adverse effects on the central respiratory control network.

Central respiratory control is finely regulated by a neural network located mainly in the ponto-medullary region of the brainstem (Richter and Spyer, 2001). In the face of hypoxic or hypercapnic challenge, maintenance of ventilatory and cardiovascular homeostasis is achieved by activation of peripheral and central chemoreceptors and subsequent modulation of this ponto-medullary respiratory control network (Marshall, 1994). It has been proposed that information from these sensory afferents is sufficient to stimulate the central respiratory control system and alter respiratory pattern independent of acidification of cerebrospinal fluid (Guyenet et al., 2017).

Pathological insults associated with central or obstructive apneas can alter chemoreceptor function, change chemoreflex integration in the central nervous system, and/or alter the properties of the central respiratory network (Gozal, 1998; Jokic et al., 2000; Harper et al., 2005; Katz et al., 2009; Carroll et al., 2010). Previous work suggests that enhanced peripheral chemoreflex activation has central effects that contribute to respiratory instability (Levy et al., 2008) and thus may play a role in perpetuating SA via creation of a positive feedback loop. Indeed, several studies have shown that increases in carotid body (CB) and central chemoreflex drive play an important role in the pathophysiology of obstructive sleep apneas (OSA) (For review see Dempsey et al., 2010).

The seminal pathological insult occurring during apneas is the repeated exposure to episodes of hypoxia-reoxygenation. This chronic intermittent hypoxia (CIH) exposure is mechanistically linked to increased peripheral chemoreflex drive, and is associated with oxidative stress and inflammation (Del Rio et al., 2011; Iturriaga et al., 2014; Iturriaga, 2017). Thus many of the major morbidities associated with SA as well as respiratory instability itself may be related to aberrant chemoreflex activation prior to and during exposure to intermittent hypoxia associated with apneic episodes. Therefore, therapies aimed at reducing chemoreflex sensitivity may be beneficial in preventing the pathophysiological sequelae of SA as well as potentially reducing the frequency of apneic episodes generated by respiratory instability.

Accordingly, several rodent models have been developed to study the pathophysiological mechanisms that contribute to enhanced peripheral and central chemoreflex drive, utilizing CIH exposure (Iturriaga et al., 2005, 2014; Rey et al., 2006; Del Rio et al., 2011). The usefulness of these models is confirmed by findings that CIH results in heightened CB activity and ventilatory chemoreflex gain in response to hypoxia (Rey et al., 2004; Del Rio et al., 2010, 2012, 2016), as well as chemoreflex-mediated increases in sympathetic activity and blood pressure (Marcus et al., 2010; Del Rio et al., 2016).

## Chemoreceptors, Respiratory Control and Cardiovascular Regulation in the Setting of Sleep Apnea

Sleep apnea syndrome is characterized by two types of events, (i) OSA and (ii) central sleep apneas (CSA). OSA is characterized by partial or complete occlusion of the upper airways during sleep; while CSA is characterized by a marked decrease in respiratory motor drive resulting from a reduction in the activity of the central respiratory network (Malhotra and Owens, 2010). It has been proposed that heightened chemoreflex gain may contribute to SA by destabilizing central respiratory network control of airway tone and/or ventilation (Del Rio et al., 2016). The process by which chemoreflex gain affects respiratory stability is often described using a control-systems engineering concept referred to as “loop gain” (Khoo, 2000).

In this application, “loop gain” can be thought of as the ratio of the size of a response (change in ventilation) to the size of a disturbance (change in PaCO_2_). The components of loop gain include controller gain, plant gain, and feedback gain. Controller gain represents the ventilatory response to PaCO_2_, plant gain represents the blood gas response to a change in ventilation, and feedback gain represents the delay associated with relaying the feedback signal (PaCO_2_) to the controller (chemoreceptors). Khoo (2000) explained that there is a chain of events, which is at the origin of ventilatory instability and attendant oscillation of ventilatory drive. Either obstructive or central apneas result in increased PaCO_2_ and activation of chemoreceptors. The duration of the oscillatory response and its magnitude are determined by the effect of ventilatory changes on PaCO_2_ (i.e., the “plant gain”), as well as by the strength of the chemoreflex response (i.e., the “controller gain”). According to this paradigm, higher loop gain is associated with greater probability of breathing instability as chemoreflex responses to changes in PaCO_2_ are likely to be disproportionate and result in ventilatory overshoots that reduce PaCO_2_ below the apneic threshold. Conversely, lower loop gains results in a more robust respiratory network which is less prone to instability and development of periodic breathing (Malhotra and Owens, 2010). With respect to the specific topics covered in this perspective article, an increase in the gain of peripheral and central chemoreceptors (controller gain) may trigger ventilatory instability and contribute to higher apnea incidence. In concordance with this notion, it has been shown in experimental low output heart failure, a condition characterized by increased apnea incidence, that peripheral chemoreceptor ablation stabilized ventilation and greatly attenuated apnea incidence (Marcus et al., 2014). In addition, selective elimination of central chemosensory neurons from the ventral medullary surface increases the apneic threshold toward eupneic ventilatory values (Takakura et al., 2008). Taken together, these studies suggest a role of both peripheral and central chemoreceptors in the development of oscillatory breathing patterns and increased apnea incidence.

### Peripheral Chemoreceptors

Peripheral chemoreceptors detect changes in arterial blood gases (mainly hypoxemia) and respond by activating the sympathetic nervous system and increasing ventilation to restore blood-gas homeostasis (Kara et al., 2003). Hypoxia-induced hyperventilation is mainly triggered by activation of the CB and to some extent by activation of the aortic body (located on the aortic arch) (Miller and Tenney, 1975; Brophy et al., 1999). The CB chemoreceptors are the main peripheral arterial chemoreceptor and are located in the bifurcation of the carotid artery. They are composed of clusters of chemoreceptor cells (type I cells) surrounded by glial cells (type II cells) (Iturriaga and Alcayaga, 2004). Type I cells are considered polymodal receptors since they respond to a wide variety of stimuli such as changes in arterial levels of pO_2_, pCO_2_, pH, blood flow, and temperature (Gonzalez et al., 1994). Upon activation by hypoxia, type I cells release ACh and ATP which interact with receptors on the sensory nerve fibers of the carotid sinus nerve (Gonzalez et al., 1994). The precise biochemical nature of the transmitter released by type I cells during hypoxic stimulation has not been completely identified since more that one molecule has been shown to be released (ACh and ATP) (Iturriaga and Alcayaga, 2004). Moreover, several peptides hormones and gasotransmitters serve as excitatory and inhibitory modulators of CB chemosensitivity (i.e., NO, histamine, and AngII) (Iturriaga and Alcayaga, 2004; Del Rio et al., 2008).

Hypoxic hyperventilation seems synchronous with the increase of the discharge frequency of the sinus nerve fibers (Vizek et al., 1987). The first central integration of sensory information from the peripheral chemoreceptors and the main areas sensory fibers from the sinus nerve project to the commissural and middle divisions of the nucleus of the solitary tract (cNTS and mNTS, respectively) (Claps and Torrealba, 1988; Finley and Katz, 1992). Neurons of the cNTS and mNTS integrate and relay information from peripheral chemoreceptors to other regions of the central nervous system to ultimately orchestrate the hypoxic hyperventilatory response (Ponikowski and Banasiak, 2001; Rosin et al., 2006; Smith et al., 2010 ). In addition, CB stimulation also triggers activation of the sympathetic nervous system to maintain adequate arterial pressure in the face of hypoxic vasodilation (Schultz and Sun, 2000). While normal CB function contributes to maintenance of blood gas homeostasis, pH regulation, and tissue perfusion, mounting evidence indicates that maladaptive changes in CB function contribute to a variety of cardiovascular and metabolic disease states (Schultz et al., 2015).

### Central Chemoreceptors

Central chemoreceptors are located mainly on the ventral surface of the medulla (Nattie and Li, 2012). In response to changes in cerebrospinal fluid CO_2_/H^+^ content, central chemoreceptor neurons send excitatory signals directly to respiratory control centers to increase breathing rate (Guyenet et al., 2005). Importantly, stimulation of central chemoreceptors also elicits an increase in sympathetic outflow mainly by their projections to pre-sympathetic control areas (Moreira et al., 2006). The precise localization of central chemoreceptors within the brain and the circuitry that is activated by CO_2_/H^+^ stimulation is still controversial. However, the retrotrapezoid nucleus (RTN) appears to play a pivotal role in the regulation of the hypercapnic ventilatory response (Guyenet and Bayliss, 2015). The RTN is mostly composed of a group of neurons that are activated by changes in cerebrospinal fluid CO_2_ and/or pH that projects to areas related to respiratory control (Lazarenko et al., 2009; Guyenet and Mulkey, 2010; Guyenet et al., 2012; Wang et al., 2013). RTN chemosensitive neurons are rhythmically active and have been shown to be activated by low pH *in vivo* and *in vitro* (Lazarenko et al., 2009; Wang et al., 2013). Interestingly, it has been shown that partial elimination of RTN chemosensory neurons (∼70%) in healthy rats increases the apneic threshold (Takakura et al., 2008), meaning apneic events occur at a higher end-tidal CO_2_. Therefore, it is plausible to hypothesize that RTN chemoreceptor neurons activity/sensitivity may contribute to ventilatory instability. In this regard, a higher ventilatory response following hypercapnia would play a major role in apnea development as enhanced CO_2_ “wash-out” would drop PaCO_2_ close to or below the apneic threshold (Topor et al., 2001). Indeed, studies in patients with heart failure have shown that apneas result in enhanced chemoreflex responses which result in a resting eupneic PtCO_2_ being closer to the apneic threshold (i.e., narrowed CO_2_ reserve, Xie et al., 2002). Furthermore, patients with heart failure that have OSA show increased ventilatory responses to hypercapnia (Solin et al., 2000). Thus, alterations in RTN chemoreceptor neuron function may contribute to apnea incidence in OSA patients by altering the apneic threshold itself or the eupneic “proximity” to the apneic threshold.

### Cellular Mechanisms of Enhanced Chemoreceptor Activity in Sleep Apnea/CIH

While the precise mechanisms underlying peripheral and/or central maladaptations to CIH are not completely understood, recent evidence suggests that reconfiguration of the neuronal network involved in sympathetic regulation and breathing stability occurs (Xu et al., 2004; Del Rio et al., 2010, 2012). Numerous studies underscore the role of oxidative stress (Marcus et al., 2010; Badran et al., 2014b; Morgan et al., 2016) and inflammation (Del Rio et al., 2010, 2012) as major drivers of augmented chemoreflex drive observed in the CIH model. Indeed, experimental CIH is associated with elevation of sympathetic outflow which is dependent on ROS production at the level of the peripheral chemoreceptors (Marcus et al., 2010; Braga et al., 2011). Taken together these studies suggest that novel treatments capable of reducing oxidative stress and/or inflammation at the level of the peripheral chemoreceptors may have potential therapeutic value for the treatment of SA-related autonomic and ventilatory dysregulation and by extension SA-related cardiovascular morbidities (i.e., systemic hypertension).

Despite numerous studies exploring the role of central nervous system ROS in cardiovascular disease, little is known about the role of ROS in the processing of the cardiovascular reflexes within the brainstem (Braga et al., 2011). CIH and Angiotensin II-derived ROS play a crucial role in the modulation of baroreceptor and chemoreceptor function, but also have been shown to play a role in altered neurotransmission in brainstem sympathetic control areas like the NTS and the RVLM (Gao et al., 2005; Nunes et al., 2010; Braga et al., 2011; Del Rio et al., 2016). To date, no studies have addressed the potential role of CIH-derived ROS on central chemoreceptor regions such as the RTN. Additional studies are needed to determine any possible contribution of RTN neurons and the central chemoreflex on the cardiovascular disturbances observed in CIH. Considering that both peripheral and central chemoreceptors potentially contribute to the pathophysiology of OSA and that oxidative stress and inflammation play important roles in abnormal chemoreceptor physiology, it is reasonable to propose that new therapeutic strategies targeting oxidative stress and inflammation may have a positive impact on aberrant peripheral and central chemoreceptor function.

## Erythropoietin and Interaction With its Receptor

Erythropoietin (Epo) is a small signaling molecule produced in the kidney and whose primary known function is the stimulation of erythropoiesis in the bone marrow (Donnelly, 2001; Dzierzak and Philipsen, 2013); however, Epo also has anti-oxidative effects. Epo directly activates intracellular anti-oxidant mechanisms such as heme oxygenase-1 and glutathione peroxidase, and Epo may inhibit iron-dependent oxidative injury indirectly by inducing iron depletion (Katavetin et al., 2007). The Epo receptor (Epo-R) is present on the surface of erythroid progenitors as a homodimer of two identical Epo-R subunits (Livnah et al., 1999), and Epo binds its receptor with very high affinity (Bunn, 2013). However, there is evidence that Epo has non-hematopoietic activity which is mediated by a ß common receptor, a heterodimer with one Epo-R monomer and CD31 (Leist et al., 2004; Chen et al., 2015). In addition to the kidney derived Epo, there is ample evidence to indicate that Epo is also produced outside of the kidney. Indeed, Epo mRNA has been detected in lungs, testis, heart, and brain in rodents (Tan et al., 1992; El Hasnaoui-Saadani et al., 2013; Pichon et al., 2016). Cells from the retina, testes, lungs, and some neurons and glial cells of the central nervous system have been shown to constitutively express several components of the Epo signaling pathway (Digicaylioglu et al., 1995; Gassmann et al., 2003; Grimm et al., 2004; Jelkmann, 2007; Yasuda et al., 2010) and expression of its target receptor (Epo-R) is found in endothelial cells, smooth muscle cells, retinal tissue, testis, and the central nervous system (Masuda et al., 1994; Ammarguellat et al., 1996; Marti et al., 1997; Bernaudin et al., 1999; Gassmann et al., 2003). Taken together, these studies suggest a role for Epo in regulation of physiological functions other than erythropoiesis (Gassmann et al., 2003). Indeed, recent data suggests that Epo regulates the control of breathing via central and peripheral actions (Soliz et al., 2005; Brugniaux et al., 2011; Voituron et al., 2014).

## Erythropoietin and Respiratory Regulation

A number of studies provide evidence that Epo plays a role in control of breathing. The Epo-R is expressed in the pre-Bötzinger complex, a key region in the brainstem involved in ventilatory rhythmogenesis and regulation (Soliz et al., 2005). Epo increases dopamine release and tyrosine hydroxylase (TH) activity in cells with neural characteristics (Masuda et al., 1994; Koshimura et al., 1999; Yamamoto et al., 2000; Tanaka et al., 2001), and Epo-R is specifically expressed in TH-positive cell groups in the brainstem (Soliz et al., 2005). Furthermore, overexpression of Epo increase brainstem catecholamine turnover in mice (Soliz et al., 2005). Interestingly, the Epo found in the central nervous system does not reach the systemic circulation due to the lack of permeability of the blood–brain barrier (Gassmann et al., 2003). These results strongly suggest that Epo-derived from the central nervous system itself must play a physiological role “*in situ*” as a local regulator of neuronal function (Jelkmann, 2007).

In addition to altering the metabolism of catecholamines in the brainstem, Epo has been shown to have similar effects in the CB, and has been shown that one injection of human recombinant Epo reduce the tidal volume during hypoxic stimulus in humans and mice (Soliz et al., 2005, 2009; Lifshitz et al., 2009). In support of this notion, recent studies have shown that Epo is released within the RVLM during hypoxic stimulation (Oshima et al., 2018), and that Epo-R is constitutively expressed in peripheral and central structures involved in ventilatory chemoreflex control (Digicaylioglu et al., 1995; Soliz et al., 2005; Lam et al., 2009; Voituron et al., 2014). Epo is known to regulate hypoxic ventilatory response (HVR) in mice by interacting with brainstem and CB (Soliz et al., 2005). The ventilatory response to hypoxia is a sex dependent response, being more pronounced in female sex (Joseph et al., 2000, 2002). Interestingly, Epo has sexually dimorphic effects on the ventilatory response to hypoxia. Indeed, it tends to increase the HVR in female mice and in women via interaction with sex steroid hormones (Soliz et al., 2009). Besides its well-known role in erythropoiesis and influence on control of breathing, Epo also exerts important cytoprotective effects.

## Erythropoietin as a Protective Molecule During Exposure to Intermittent Hypoxia

**FIGURE 1:**
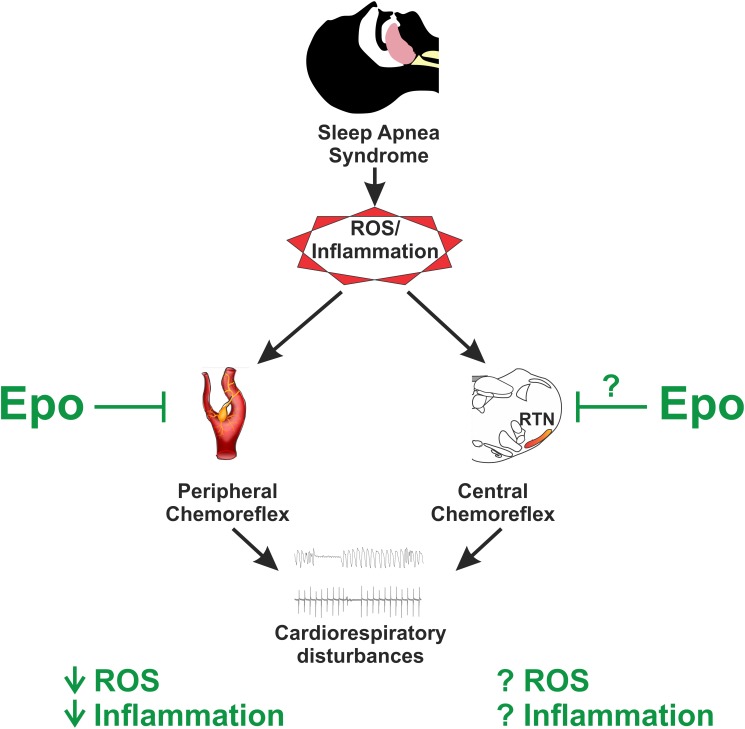
Potential role of erythropoietin (Epo) on sleep apnea pathophysiology. Sleep apnea syndrome is characterized by augmented ROS and inflammatory molecules, which play a pivotal role on the pathophysiology by triggering augmented chemoreflex sensitivity which ultimately lead to altered breathing and cardiovascular events. Epo has been shown to reduce both ROS and inflammation in the brain and at peripheral chemoreceptors located primarily at the carotid body; however, its role on central chemoreceptors sensitivity remains to be studied. Thus, it is plausible to hypothesize that Epo administration and/or its derivates in the setting of sleep apnea may offer an anti-oxidant/inflammatory therapy to control for the augmented chemoreflex drive.

It has been shown that Epo exerts a neuroprotective role in several diseases due to its anti-apoptotic (Sirén and Ehrenreich, 2001), anti-cytotoxic (Morishita et al., 1997), anti-oxidative (Koshimura et al., 1999), and anti-inflammatory (Villa et al., 2003) properties. Increases in oxidative stress and inflammation are recognized as key mediators affecting control of breathing and cardiovascular function following exposure to CIH in rodents (Del Rio et al., 2010, 2011, 2012, 2016; Marcus et al., 2010; Iturriaga et al., 2014). It has been shown that CIH induces oxidative stress in the CB and potentiation of CB-mediated chemoreflex drive (Del Rio et al., 2010; Marcus et al., 2010). In addition, increased expression of pro-inflammatory cytokines in the CB has been shown following exposure to CIH (Del Rio et al., 2011, 2012). Furthermore, we showed that ibuprofen treatment selectively reduces central inflammation in the NTS in rats exposed to CIH, and that ibuprofen treatment decreases the ventilatory response to hypoxia (Del Rio et al., 2012). Taken together, these results suggest that oxidative stress and inflammation acting predominantly on chemoreflex pathways are involved in the altered chemoreflex function and attendant autonomic dysregulation following CIH. Thus, it is plausible that administration of Epo could have a positive effect on control of breathing and autonomic function during/after exposure to CIH (**Figure 1**).

## Conclusion

Sleep apnea syndrome, characterized by cyclic and repeated exposure to brief episodes of hypoxia and hypercapnia, is recognized as a major public health problem worldwide. SA can occur as a result of upper airway obstruction and/or as a result of abnormal respiratory control resulting from aberant peripheral and central chemoreflex function. Currently, there are no treatments that specifically target abnormal control of breathing in SA. Epo has recently been shown to have novel neuroprotective properties associated with anti-oxidant and anti-inflammatory effects. Increases in oxidative stress and inflammation are both recognized as key mediators in respiratory and cardiovascular disturbances following exposure to CIH. Accordingly, we propose that future studies should address the potential beneficial effect of Epo or Epo-like compounds on cardio-respiratory function during or after exposure to CIH. Epo-induced erythropoiesis could be detrimental in patients with SA therefore, developing new Epo-derived compounds that can bind to the Epo-R with little or no effect on erythropoiesis would be optimal in terms of therapeutic value.

In summary, uncovering a role for Epo in the regulation of the ventilatory response to hypoxia and/or hypercapnia as well as ventilatory instability will open new avenues in the field of control of breathing in the pathological setting of SA. Furthermore, determining the potential therapeutic efficacy of Epo or Epo-derived compounds on the enhanced chemoreflex sensitivity observed during CIH will be of potential therapeutic value.

## Author Contributions

All authors have approved the final version of the manuscript and agree to be accountable for all aspects of the work. All persons designated as authors qualify for authorship, and all those who qualify for authorship are listed.

## Conflict of Interest Statement

The authors declare that the research was conducted in the absence of any commercial or financial relationships that could be construed as a potential conflict of interest.
